# *Rhodotorula* spp. in Laboratory and Veterinary Clinical Practice: Contamination or an Emerging Problem?

**DOI:** 10.3390/ani15223299

**Published:** 2025-11-15

**Authors:** Kacper Wykrętowicz, Ewelina Czyżewska-Dors, Arkadiusz Dors, Małgorzata Pomorska-Mól, Agata Augustyniak, Dominik Łagowski

**Affiliations:** 1 Department of Preclinical Sciences and Infectious Diseases, Poznan University of Life Sciences, Wołyńska 35, 60-637 Poznań, Poland; kacperwykretowicz8@gmail.com (K.W.); adors@up.poznan.pl (A.D.); malgorzata.pomorska@up.poznan.pl (M.P.-M.); agata.augustyniak@up.poznan.pl (A.A.); 2 Department of Internal Diseases and Diagnostics, Faculty of Veterinary Medicine and Animal Science, Poznan University of Life Sciences, 60-637 Poznań, Poland; ewelina.czyzewska-dors@up.poznan.pl

**Keywords:** *Rhodotorula* spp., microbiological diagnostics, opportunistic fungi, resistance, fungal infection, otitis externa

## Abstract

*Rhodotorula* spp. are ubiquitous red-pigmented yeasts increasingly reported as opportunistic animal pathogens. Recognition matters because underdiagnosis can misguide therapy, especially in companion-animal otitis externa. This review supports laboratory and clinical decisions by summarising taxonomy and ecology, host risk factors, diagnostics, virulence factors, antifungal susceptibility, and veterinary cases. This review addresses: (1) taxonomy and ecology; (2) clinical epidemiology and predisposing factors (immunomodulation, prior antibacterial therapy, chronic inflammation); (3) diagnostics—cytology, organism burden, repeat or pure culture, MALDI-TOF MS, ITS sequencing—with a brief comparison of feasibility in veterinary practice; (4) virulence factors—adhesion and biofilm on abiotic surfaces, hydrolytic enzymes, capsule in some strains, haemolysins, urease, and carotenoids that protect against oxidative stress; (5) antifungal susceptibility and therapy—intrinsic resistance to echinocandins, often high azole MICs, and the most consistent in vitro activity of amphotericin B ± flucytosine; and (6) a synthesis of veterinary case reports. *Rhodotorula* spp. should not be dismissed as contaminants when clinical signs match laboratory evidence; distinguishing infection from colonisation requires clinicomicrobiological correlation. This review highlights the need for standardised susceptibility testing and veterinary breakpoints, prospective data on burden and outcomes, better data on biofilm behaviour on clinical materials, environmental surveillance, and practical diagnostic and treatment guidance.

## Simple Summary

Red–orange yeast-like fungi, such as *Rhodotorula* spp., are often dismissed as harmless laboratory contaminants. However, in certain situations—such as long-standing ear disease in dogs and cats, a weakened immune system, or after prolonged antibiotic use—they can contribute to illness. These fungi can cling to skin and medical devices, build biofilm that help them persist, and release enzymes that injure tissues. Several commonly used antifungal drugs are ineffective against them, whereas amphotericin B, sometimes combined with flucytosine, is typically effective in treating severe infections. In everyday practice, determining whether a positive culture indicates actual infection or mere colonisation requires linking the test results with the animal’s signs, for example, by examining ear smears under the microscope, assessing the extent of growth, repeating cultures, and utilising modern identification methods when necessary. This review brings together current knowledge on where *Rhodotorula* spp. lives, how to recognise it, why it occasionally causes disease, which treatments are likely to help, and what has been reported in animals to help veterinarians target therapy and avoid unnecessary medicines.

## 1. Introduction

Otitis externa (OE) is among the most common health problems in companion animals, and in dogs, it is one of the leading reasons for veterinary visits [1,2]. In European populations, OE is estimated to affect 9% to 20% of dogs; in the United Kingdom, a one-year prevalence of 10.2% was reported, making it the most commonly recorded condition [2,3]. This disorder is not only prevalent but also associated with substantial discomfort—OE causes pain and pruritus, reduces animal welfare, and may be classified among high-burden conditions [4,5]. OE is multifactorial: its development involves predisposing factors (e.g., external ear canal anatomy, allergic skin disease, foreign bodies, trauma), inciting (primary) causes of inflammation, and perpetuating factors that maintain the inflammatory process [5,6]. In the active phase, signs related to overgrowth and disruption of the ear microbiota often predominate—namely, excessive proliferation of microorganisms, especially bacteria and yeast-like fungi [5,7]. Effective treatment and prevention of recurrence require the simultaneous recognition and control of all these elements—particularly identification and therapy of the primary cause that initiated the disease process [5,8].

In clinical practice, *Malassezia pachydermatis* is the yeast-like fungus most commonly identified in canine and feline OE [9,10]. In addition to *Malassezia*, cultures from the external ear canal occasionally yield other yeasts—*Candida* spp., *Trichosporon* spp., *Cryptococcus* spp., and *Rhodotorula* spp.—typically as mixed growth with other microorganisms [11,12,13,14]. The contribution of these less frequently encountered fungi is often underestimated, as their presence is commonly interpreted as contamination or clinically irrelevant colonisation [5,9]. While such caution is understandable, it may lead to overlooking their etiologic role [15,16]. Under certain conditions—such as chronic inflammation, impairment of host defence mechanisms (immunomodulation, immunosuppression), or prior antibacterial therapy—even these seemingly saprophytic fungi can participate in the pathogenesis of symptomatic inflammatory disease [17,18,19]. Therefore, awareness of the full spectrum of yeasts reported in OE—even when isolations are rare—has practical diagnostic and therapeutic value [5].

*Rhodotorula* spp. are red-to-orange–pigmented, yeast-like fungi that are widely distributed in the environment, inhabiting diverse niches (soil, water, plants, damp surfaces) and—as potential commensals—the skin and mucous membranes of animals and humans [20,21,22]. The species most frequently reported in opportunistic infections are *R. mucilaginosa* and *R. glutinis* [13,23,24]. Although long regarded as low-level pathogenicity organisms, current epidemiological and clinical analyses classify *Rhodotorula* spp. as opportunistic pathogens, particularly in hosts with impaired immunity [25]. In humans, infections are often associated with biomaterials, intravascular catheters, and generalised fungemia. In contrast, case reports in veterinary medicine are less common across species. However, they are documented, including isolations from the nasal passages and external ear canals of dogs and cats with clinical signs [13,25].

Available data indicate that fungi from genus *Rhodotorula* possess several virulence factors, including biofilm formation on surfaces (including biomaterials), adhesion, and secretion of hydrolytic enzymes (e.g., phospholipases and aspartyl proteases) [22,26,27,28]. These attributes are variable and their expression depends on environmental conditions [26,29]. Experimental models and in vitro studies show that *R. mucilaginosa* forms biofilm with properties that hinder eradication and can display enzyme expression profiles potentially relevant to tissue invasion [27,29]. Consequently, distinguishing colonisation from infection requires clinical-microbiological correlation, including cytology, assessment of growth burden, isolation in pure culture from lesional material, repeat isolation, and molecular testing [9,30,31].

From a treatment standpoint, *Rhodotorula* spp. are intrinsically non-susceptible to echinocandins and exhibit high MIC values to many azoles, particularly fluconazole. Amphotericin B, sometimes combined with flucytosine, shows the most consistent in vitro activity and has been documented to have clinical effectiveness in humans. EUCAST/CLSI does not establish clinical breakpoints for *Rhodotorula* species; so, MIC interpretation relies on MIC distributions [32,33,34,35]. For *Rhodotorula* spp., validated, species-specific interpretive criteria for disk diffusion are also lacking. CLSI M44 applies only to selected *Candida* spp., while EUCAST provides a reference MIC method (E.DEF 7.4) and does not publish a disk diffusion method [36,37]. In practice, disk diffusion may be chosen for cost reasons; however, for *Rhodotorula* spp., the absence of breakpoints means that zone diameters should not be categorised (S/I/R) and may not accurately reflect the susceptibility profile [36,38]. In veterinary medicine, organism-specific therapeutic standards for *Rhodotorula* have not been developed; reports suggest an approach guided by isolate-level MIC testing and cautious extrapolation from human medicine (amphotericin B ± flucytosine), together with topical therapy and modification of the ear environment [5,39].

This narrative review aims to organise scattered information on the *Rhodotorula* spp. species in the context of its significance for otitis externa. Presenting the full spectrum of clinical symptoms attributed to the genus *Rhodotorula* is intended to facilitate differential diagnosis and optimise the treatment of ear infections. The collection and systematisation of this data, together with a summary of available descriptions of various veterinary cases (not only related to ear infections) involving these fungi, should aid clinical decision-making in complex, rare cases of OE caused by these yeasts.

## 2. Taxonomic Classification and Revision

The genus *Rhodotorula* was established in the late 1920s by the Canadian microbiologist Francis *C.* Harrison, who delineated a group of yeasts characterised by pink colony pigmentation (the name *Rhodotorula* derives from the Greek *rhodon*, meaning “pink”) [20,40,41]. In the subsequent decades, numerous newly described species were gradually assigned to *Rhodotorula*, totalling more than 100 [20]. The literature also included names now regarded as synonyms, such as *Chromotorula* (also established by F.*C.* Harrison in 1927) and *Rhodosporidium* (the teleomorph described by I. Banno in 1967) [42]. Currently, in accordance with the One Fungus–One Name principle, these historical names for the respective taxa have, for the most part, been consolidated under *Rhodotorula*, which retains priority as the older and more widely used name [20,43,44].

Multigene phylogenetic analyses showed that the broadly defined genus *Rhodotorula* was polyphyletic, artificially grouping species with different evolutionary origins [20]. Fungi traditionally assigned to *Rhodotorula* spp. in fact belong to several phylogenetically distant lineages within the *Basidiomycota*, despite morphological similarities, such as carotenoid pigments, that give colonies a red-to-pink colouration [20,45].

As a result of the above findings, a thorough revision of the systematics of these fungi was carried out [46]. To distinguish monophyletic lines reflecting actual genetic relationships, a significant portion of the species formerly classified in the genus *Rhodotorula* were transferred to other genera, often placing them in completely separate families and orders within the *Basidiomycota* [43,46]. For example, based on new taxonomic approaches, some species previously excluded from *Rhodotorula* have been classified into orders such as *Cystobasidiales*, *Tremellales*, and *Trichosporonales* [43,46,47]. These changes aimed to split the former polyphyletic classification of *Rhodotorula* fungi and assign individual species to their appropriate monophyletic taxa, in accordance with the results of DNA analyses [46].

The consequence of the above revisions is a limitation of the definition of the genus *Rhodotorula* to a relatively small group of species closely related to the type species, i.e., *Rhodotorula glutinis* [48]. Currently, *Rhodotorula sensu stricto* constitutes a monophyletic clade comprising at least 15 species of yeast-like fungi [47,48]. Representatives of this limited definition of the genus include species, such as *Rhodotorula alborubescens*, *R. araucariae*, *R. babjevae*, *R. dairenensis*, *R. diobovata*, *R. evergladensis*, *R. glutinis*, *R. graminis*, *R. kratochvilovae*, *R. mucilaginosa*, *R. pacifica*, *R. paludigena*, *R. sphaerocarpa*, *R. taiwanensis,* or *R. toruloides* [40,47]. These fungi preserve the typical morphological and physiological characteristics historically described for the genus *Rhodotorula*: they produce carotenoid pigments that colour their colonies pink or orange, they reproduce asexually by budding, and in forms with sexual stages in their life cycle, they form characteristic teliospores [20,49].

As a result of phylogenetic studies, fungi formerly described as *Rhodotorula rubra* are now classified as *Rhodotorula mucilaginosa* [34,40]. The name *R. rubra* fell out of use after analysis of typical strains, as it was shown that the reference strain *R. rubrarubra* is, in fact, the strain *R. glutinis*. At the same time, the type *R. mucilaginosa* turned out to be identical to the former *R. rubra*. As a result, the name "*mucilaginosa*" was kept for this species. Similarly, the species *Rhodotorula minuta*, once considered separate, was later recognised as identical to *R. mucilaginosa*, only to be transferred to the genus *Cystobasidium* as *Cystobasidium minutum* [43]. *Rhodotorula pilimanae* was also recognised as a synonym of *R. mucilaginosa* [40]. In older literature, the name *Rhodosporidium* was used for the sexual stages of fungi included in the genus *Rhodotorula*; however, after reclassification, the former *Rhodosporidium toruloides* has the valid name *Rhodotorula toruloides* [20]. Due to the standardisation of naming rules (One Fungus = One name), the use of the name *Rhodotorula* for the monophyletic approach to the genus has been established. All the systematic changes have ensured consistency with the current state of phylogenetic knowledge—*Rhodotorula* remains a clearly distinct taxon within the *Basidiomycota*, in line with actual genetic relationships [20].

## 3. Key Aspects of Identifying Yeast-like Fungi of the Genus *Rhodotorula*

The diagnosis of infections caused by fungi of the genus *Rhodotorula* is based on classical microbiological methods (microscopic examination and culture), supplemented by biochemical tests [25,30]. Modern identification techniques, such as MALDI-TOF mass spectrometry or molecular methods based on DNA analysis, are also increasingly used [50,51]. Unlike some dimorphic fungi or moulds, serological tests are not routinely used in the diagnosis of *Rhodotorula* spp.—there are no specific tests for detecting antigens or antibodies of these fungi [30,35]. Furthermore, non-specific immune responses (e.g., the latex antigen test for *Aspergillus* spp.) may yield false-positive results if the sample is contaminated [52].

### 3.1. Culture

**Figure 1:**
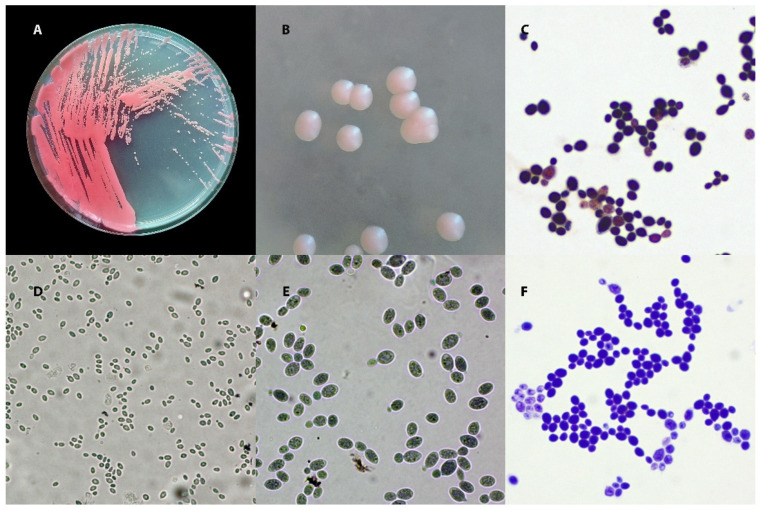
Macro- and micromorphology of *Rhodotorula mucilaginosa*. (**A**) Growth on Sabouraud Dextrose Agar (SDA) after 3 days at 30 °C. (**B**) Close-up of individual colonies. (**C**) Gram-stained preparation, 1000×. (**D**,**E**) Chlorazol Black E-stained preparation: (**D**) 400×; (**E**) 1000×. (**F**) Diff-Quik-stained preparation, 1000×.

Fungi from genus *Rhodotorula* colonies multiply on standard fungal media (usually appearing within 2–5 days on Sabouraud agar) [13,40]. They have a characteristic smooth surface—colonies may be shiny, soft, moist, and sometimes slightly slimy [53]. These fungi produce carotenoid pigments that give the colonies a colour ranging from salmon and pink to coral and orange-red [53]. The intensity of pigmentation depends on the species: *R. mucilaginosa* usually colours more intensely (reddish colonies), while *R. glutinis* more often forms salmon-coloured (pale pink) colonies [40,53] (Figure 1). In liquid cultures (e.g., in Sabouraud broth), *Rhodotorula* spp. yeasts can produce a coloured sediment and a thin film on the surface of the liquid; this phenomenon depends on the strain and incubation conditions [13].

### 3.2. Microscopic Examination

In direct and culture preparations, the cells of these fungi are visible as spherical to oval (blastoconidia) [53] (Figure 1). These yeast-like fungi do not form true mycelium or chlamydospores, and pseudohyphae are generally absent (in a few cases, only residual forms have been described) [54,55]. The cells are usually several micrometres in diameter (the range depends on the strain and medium; values of 2.5–14 µm have been described) and are Gram-positive [13,53]. Some cells may have a mucous envelope, which can be indirectly visualised by negative staining (nigrosine, Chinese ink) [56,57]. On media that stimulate hyphae formation (rice agar), *Rhodotorula* spp. give a negative result in the filamentation test, which distinguishes them from *Candid*a *albicans/dubliniensis* [40,53,58].

### 3.3. Biochemical Tests

Due to the high morphological similarity of individual *Rhodotorula* species, their identification is primarily based on physiological characteristics (biochemical profiles) [58]. These fungi do not ferment sugars (they exhibit oxidative metabolism), but can assimilate many simple sugars and alcohols as a source of carbon [53]. Species of fungi of the genus *Rhodotorula* are generally urease-positive [58,59,60]. In laboratory practice, commercial test kits such as API 20C AUX or ID 32C (bioMérieux; software version 1.3.1) are used for identification, which allow the genus *Rhodotorula* to be recognised and, with a complete biochemical profile, the species to be determined [61,62]. However, it should be emphasised that conventional species identification can be difficult – the accuracy of such tests is sometimes limited and the results are not always fully reproducible [63]. The standard test kit includes, among other things, the ability to assimilate various carbon sources (e.g., glucose, maltose, xylose, lactose, melibiose, sugar alcohols), nitrogen assimilation from nitrates, the presence of the enzyme catalase, pigment production on specific media, and the ability to grow at different temperatures [53,58,64].

The two most frequently isolated species—*R. mucilaginosa* and *R. glutinis*—show subtle biochemical differences that allow for laboratory differentiation [25,35]. *R. mucilaginosa* usually grows at 37 °C (and even up to 40 °C), whereas growth at 37 °C is variable for *R. glutinis* [53]. Both species produce carotenoid pigments; *R. mucilaginosa* often assimilates the five-carbon sugar D-xylose, while xylose use is variable in *R. glutinis* [53]. Both yield positive urease tests, which helps distinguish them from *Candida* spp. [53]. Nitrate assimilation does not reliably distinguish the species: *R. mucilaginosa* is typically KNO_3_ (−), and *R. glutinis* is often also KNO_3_ (−) in clinical panels. However, some studies have detected nitrate reductase in selected *R. glutinis* strains [53,65]. When identification is uncertain—and given the variability across sources—adding a nitrate assimilation test is recommended. In one study, the inclusion of KNO_3_ in the biochemical panel increased the accuracy of *Rhodotorula* identification to 90.3% compared to the reference method [26]. Clinically, identification at the genus level is often sufficient for preliminary decisions; however, where possible, species-level testing should be performed and the results confirmed if they are inconclusive [32,35].

### 3.4. Differentiation from Other Species of Yeast-like Fungi

In laboratory practice, mycological cultures are often conducted at 28–32 °C for 3–5 days [58,66]. These conditions favour the biosynthesis of carotenoids and the growth of so-called ‘red yeasts’, whose colonies may macroscopically resemble *Rhodotorula mucilaginosa* or *R. glutinis*. At the same time, the intensity of pigmentation in many yeast-like fungi species decreases significantly at 37 °C, which is why colour assessment alone after incubation at a higher temperature may be insufficient [67,68]. Fungi of the genus *Rhodotorula* should be differentiated primarily from fungi such as *Sporobolomyces*, *Cystobasidium*, *Cystofilobasidium*, *Xanthophyllomyces dendrorhous*, and *Metschnikowia pulcherrima* [69]. *M. pulcherrima* produces pulcherrimine (an iron-chelating pigment), which causes reddish-brown zones around colonies on Fe^3+^-containing media—a feature not present in *Rhodotorula* [70]. Many pigmenting fungi grow poorly or not at all at 37 °C, whereas *R. mucilaginosa* can grow at this temperature [53,71,72].

Unlike *Rhodotorula* spp., *Candida* species generally do not produce carotenoids and may tend to form pseudohyphae [40,53]. *C. albicans* and *C. dubliniensis* are characterised by a positive filamentation test, which remains negative in *Rhodotorula* spp. [53,58]. It is also crucial to distinguish *Rhodotorula* from *Cryptococcus neoformans/gattii* complex. Both genera are usually positive in the urease test, and the colonies may be slimy (with a capsule). However, the *Cryptococcus neoformans*/*gattii* complex produces melanin, the presence of which can be observed on agar with niger seed or sunflower seed extract, giving the colonies a brown-black colour [56,73]. *Cryptococcus* fungi typically assimilates myo-inositol, which practically differentiates it from *Rhodotorula* species [53]. A summary of the main identifying features is presented in Table 1.

### 3.5. Automatic and Molecular Methods

Nowadays, the identification of yeast-like fungi is more frequently based on MALDI-TOF MS mass spectrometry, due to the simplicity, speed and accuracy of this method [74,75]. However, its effectiveness depends on the quality and scope of the reference spectrum library available in a given system; gaps in the library may lead to ambiguous or erroneous results; therefore, it is recommended—in accordance with CDC/UK SMI guidelines—to use a complete protein extraction protocol and repeat the test, and in case of persistent uncertainty, to switch to molecular methods [50,76].

In doubtful cases or for research purposes, the standard is to sequence the ITS regions (the primary identification marker for fungi) or the D1/D2 domains of LSU rDNA; the sequences obtained should be compared with already deposited sequences (e.g., NCBI), and the current taxonomy and types should be verified in dedicated repositories (MycoBank/Index Fungorum). PCR/sequencing techniques remain crucial for describing new species and conducting taxonomic revisions [51,77,78,79,80].

### 3.6. An Integrated Approach to the Diagnosis of Rhodotorula spp. Infections

In cases of infection limited to a specific location (e.g., skin lesions, infection at the site of vascular catheter insertion, non-healing infection of the external ear canal), it is recommended to simultaneously collect clinical material for mycological and histopathological examination [31,81]. In that case, a biopsy should be performed on the lesion – part of the collected material should be preserved in formalin for histopathological examination, and part should be immediately cultured on media [31,81]. This dual-track approach increases the chance of detecting the pathogen and correctly interpreting the result. The culture will provide a strain of fungus for species identification and ASFT, while histopathological examination will show fungal structures in the tissue and signs of invasion [31].

The detection of fungal elements in situ confirms that we are dealing with a real infection, rather than surface colonisation or sample contamination [81]. Basic histopathological examination (H&E staining) and additional staining (PAS or Gomori silver staining) allow for differentiation between infection and colonisation based on the evidence of fungal elements in the tissue and the host inflammatory response [81]. Significantly, histopathology results can be obtained comparatively quickly—typically within 7–14 days —and in fast-track mode within 24–48 h; however, in veterinary cases, this option is rarely possible [81]. Mycological culture, on the other hand, requires more time. The collected samples or swabs should be inoculated onto basic fungal media (Sabouraud agar, CandidaChrom aagar) as soon as possible and incubated at 25–30°C. The colonies of *Rhodotorula* spp. may appear after 2–3 days of incubation on agar, especially if the inoculum is dense and comes from sites with heavy fungal colonisation [22]. However, if no growth is observed within the 5 days, this does not exclude the presence of the pathogen in the sample. As in the diagnosis of fungaemia, the use of an alternative medium (Potato Dextrose Agar) and a more extended incubation period can reveal the presence of *Rhodotorula* spp. [82]. Therefore, when there is a high clinical chance of infection, it is recommended to extend the culture to 14 days [82]. In the meantime, in addition to histopathological examination, cytological methods can be used on fresh material [83]. In the case of superficial lesions (e.g., on the skin or in the ear canal), a simple cytological smear stained with a method such as Diff-Quik or Gram can reveal yeast-like cells on the same day [9]. In that case, the detection of numerous budding yeast cells in a cytological smear allows treatment to be initiated on the same day rather than delaying treatment for several days [9].

When is suspected disseminated fungal infection caused by yeast-like fungi (e.g., fungaemia, CNS involvement), rapid detection and identification of the pathogen (preferably to the species level) are crucial [84]. In human patients, it is recommended to immediately collect at least 2, usually 2–3, sets of blood cultures, each containing 20–30 mL (40–60 mL in total), to increase detection sensitivity [85]. In small animals, large volumes are usually impossible to collect; therefore, unlike the recommendations for adult humans (20–30 mL per set), smaller volumes and paediatric bottles can be used in veterinary diagnostics [86]. However, it may be necessary to limit the number of collections for welfare reasons; ≥2 samples should be obtained whenever possible [86,87]. Unfortunately, this may result in reduced sensitivity—a smaller sample volume reduces the chance of detecting a rare pathogen in the blood [86,88]. Nevertheless, as in humans, it is recommended to collect at least two blood samples from different sites and at different times, if possible, to distinguish true fungaemia from skin contamination during collection [87,89].

Automatic blood culture systems (e.g., BACTEC, BacT/ALERT) usually signal microbial growth within a few days of sample inoculation [90]. In the case of fungi of the genus *Candida*, a positive signal is often obtained after 2–3 days, but rarer species may require longer incubation [82,91]. It has been reported that *Rhodotorula mucilaginosa* fungemia showed growth only around the fifth day of culture [82]. For this reason, both in human and veterinary medicine, the incubation of standard bottles is not routinely extended beyond 5 days; if suspicion persists, mycological bottles are added or complementary methods are used [92,93]. At the same time, rapid antigen tests can be performed on serum; for example, in humans, the level of 1,3-β-D-glucan is measured, which can quickly confirm the presence of fungal wall components in the blood [94,95]. However, it should be remembered that *Rhodotorula* spp. does not have a species-specific antigen test, and β-D-glucan test results may be falsely low for this pathogen despite infection [96]. Nevertheless, a positive test result (if present) can provide a valuable early indication of a fungal infection, justifying the implementation of empirical therapy [94]. In veterinary practice, tests such as β-D-glucan are not routinely used—diagnosis is usually based mainly on culture and microscopic examination [13,97]. However, in justified cases, reference laboratories can be used (e.g., for tests intended for humans), bearing in mind that results in animals should be interpreted with caution.

In the absence of MALDI-TOF, rDNA marker sequencing (ITS and/or LSU D1/D2) is used, usually in a reference laboratory [78]. Sequencing provides high species resolution but takes longer than MALDI TOF (due to additional time for transport/DNA isolation/PCR/sequence analysis) [78]. This situation is more common in veterinary diagnostics, where not all laboratories have a MALDI-TOF system or biochemical panels for yeast-like fungi – in practice, samples from animals are often sent to specialised centres, which prolongs the time to final diagnosis [98,99]. In practice, for veterinary patients, full results (ID ± AFST) often exceed 7 days, and for shipping/sequencing, they can reach 10–14 days. In contrast, in modern human hospital laboratories, the species is often known within 24 h of obtaining the colony, and with direct MALDI-TOF identification from a positive bottle, even within a few dozen minutes [97,99]. A summary of the main advantages, limitations, time-to-results, and typical applications is presented in Table 2.

## 4. Virulence Factors of *Rhodotorula* spp. and Their Significance in Pathogenesis

### 4.1. Pathogenesis

Infections caused by *Rhodotorula* spp. are opportunistic, as they primarily develop in hosts with impaired immune responses or a disturbed microbiota balance [13,35,110]. Under normal immune conditions, these yeast-like fungi are rarely able to overcome the body’s defences and cause disease [13,25]. The pathogenesis of rhodotorulosis resembles that of cryptococcosis or *Candid*iasis in some aspects [13,56]. The primary route of entry into the body is most likely transdermal—the yeasts colonise the skin or enter the body from the environment through medical implants (such as vascular catheters, peritoneal catheters, and contact lenses), then break through the skin/mucosal barrier and enter the bloodstream [54,110,111]. In the case of contamination of sterile fluids (e.g., parenteral nutrition preparations), fungal cells can be introduced directly into the bloodstream via the vascular route [112,113]. An alternative route is inhalation, but there is no evidence to support this form of infection. Instead, inhalation may lead to colonisation of the respiratory tract in individuals with permanent respiratory problems or those who are chronically hospitalised, but full-blown pneumonia is rare [21,114].

**Figure 2:**
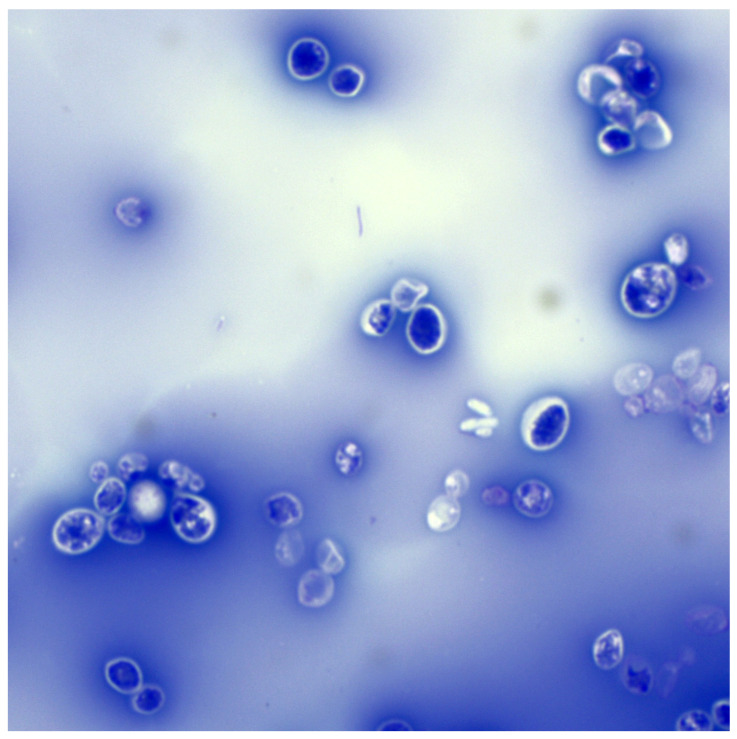
*Rhodotorula mucilaginosa*—capsules. Preparation stained with Loeffler’s blue; magnification 1000×.

After invading the host’s blood and tissues, *Rhodotorula’s* cells are subjected to innate immune mechanisms. Neutrophils, monocytes and macrophages play an essential role in controlling and spreading these fungi [115]. Phagocytes recognise cells through receptors for mannan and glucan (TLR, Dectin), among others, although the polysaccharide capsule in some *Rhodotorula* strains may mask specific epitopes and hinder phagocytosis [57,116]. Nevertheless, most cells are phagocytosed and killed inside phagolysosomes due to the secretion of reactive oxygen species and proteolytic enzymes [117]. The previously mentioned antioxidant defence capacity is significant here – carotenoids can partially neutralise free radicals, which potentially increases the survival rate of yeast inside macrophages [118,119]. However, if the host has a properly functioning immune system, efficient neutrophils quickly eliminate fungal cells before they multiply excessively [25]. In individuals with neutropenia or phagocyte dysfunction (e.g., in the course of diabetes or corticosteroid treatment), fungal cells can avoid destruction and (most likely) survive inside macrophages, similar to *Cryptococcus neoformans* [25]. Some fungi of the genus *Rhodotorula* are capable of growing at 37 °C and are tolerant to oxidative stress, which enables them to survive in the human or animal body [13,21,26]. Furthermore, in the presence of serum, some *R. mucilaginosa* cells produce a (macro)capsule (Figure 2) that, although much thinner than that of *Cryptococcus neoformans*, remains clearly visible in routine light microscopy. This capsule can limit the access of antibodies and complement to the fungal wall, thereby preventing opsonisation and phagocytosis [57].

In the development of fungemia, the immune system activates mechanisms similar to those in *Candid*emia [115]. This leads to the activation of the alternative complement pathway, the inflow of neutrophils to the sites of infection, and the production of pro-inflammatory cytokines (TNF-α, IL-6, GM-CSF) that stimulate phagocytosis [120]. Interestingly, laboratory tests used for invasive *Candid*iasis may give different results in rhodotorulosis [30,35]. For example, the level of the cell wall antigen 1,3-β-D-glucan in the blood is negative in patients with *Rhodotorula*-induced fungemia, despite the presence of an active infection [121]. A case of catheter-associated fungemia caused by *R. mucilaginosa* has been described in a patient whose β-D-glucan test remained negative [121]. This is explained by the fact that fungi belonging to *Basidiomycota* may release less of this antigen into the blood or have a wall that is less reactive in this test [122]. On the other hand, tests detecting mannan (*Candid*a spp. antigens) are not applicable, as *Rhodotorula* spp. mannan is different and is not detected by standard kits [84,123]. The humoral response against *Rhodotorula* spp. has not been well characterised; it is believed that anti-glycoprotein antibodies may facilitate phagocytosis (opsonisation), but they are not decisive for eliminating infection in the absence of efficient phagocytes [115,124].

In healthy individuals, defence mechanisms such as physical barriers and phagocytosis by neutrophils usually effectively eliminate *Rhodotorula* cells before they cause infection [115]. However, in immunocompromised individuals, even the comparatively low virulence of *Rhodotorula* spp. may be sufficient to cause severe, generalised infection [110]. The presence of catheters and medical implants promotes the growth of these fungi; they provide a surface for colonisation and bypass the skin barrier. The clinical symptoms of rhodotorulosis result from a combination of host immune system failure and the inherent potential of the fungus itself [21]. Understanding the virulence factors of *Rhodotorula* spp. is crucial for developing more effective strategies to prevent and treat these rare but serious infections [21,25].

### 4.2. Virulence Factors

*Rhodotorula* spp. are not classified as a highly virulent fungi, but rather as an opportunistic pathogens [110,125]. Nevertheless, studies have shown that these fungi possess several virulence factors analogous to those found in *Candid*a *albicans* and other fungi [38,126]. The most important of these include the ability to form biofilms on abiotic surfaces, the production of hydrolytic enzymes (e.g., phospholipases, proteases, lipases), the presence of carotenoid pigments that can protect cells, the ability to adhere to cells and medical devices, the ability to lyse erythrocytes and, in some strains, the production of a polysaccharide capsule and the formation of pseudohyphae [125,127]. It is worth noting that the expression of virulence factors among *Rhodotorula* spp. strains is variable, with considerable diversity between strains in terms of their pathogenicity determinants [126,127].

#### 4.2.1. Biofilm Formation

The ability to form biofilms on surfaces (such as plastic catheters) is a recognised virulence factor of yeast-like fungi [128,129,130]. The biofilm is a three-dimensional structure of microbial colonies, submerged in an extracellular matrix, that is highly adhesive, making it difficult for antifungal agents to penetrate [128,131]. *Rhodotorula* spp. show the ability to form biofilms, although generally to a lesser extent than *C. albicans* [126]. In vitro, most *R. mucilaginosa* strains tested formed a thin biofilm on polystyrene plates at 37 °C (on a serum medium); however, the amount of biomass after 72 h of incubation was significantly lower than that of the comparative *C. albicans* strains [126]. A study conducted by Seifi et al. found that only about 23.5% of isolates of various *Rhodotorula* species produce a distinct biofilm, and the best conditions for its formation are a temperature of 25 °C and 72 h of incubation [127]. This suggests that the ability to form biofilm may vary between species—isolates of *R. glutinis* and *R. minuta* formed biofilm less efficiently than *R. mucilaginosa*, which is a species capable of growing at 37 °C and in the presence of serum [126,127]. Despite its relatively weak ability to form biofilm, its presence on long-term vascular catheters may explain the reported cases of late-onset fungaemia–yeasts can exist for some time in the form of biofilm on the surface of the catheter [132,133] Although uncommon, human cases of *Rhodotorula mucilaginosa* endocarditis are well documented on both native and prosthetic valves, typically in the context of intravascular devices or immunosuppression; mixed infections have also been described [134,135]. Persistent fungaemia frequently resolves only after catheter removal and amphotericin-based therapy, and valve surgery is sometimes required. In keeping with the organism’s ability to form biofilms, intravascular infection is predominantly device- or prosthesis-associated; biofilm formation on intact native endothelium during transient fungaemia is not clearly demonstrated and appears uncommon [25,110]. Biofilm also acts as a barrier to treatment – fungal cells in biofilm show increased resistance to antifungal agents, which hinders the eradication of colonies [27,38].

#### 4.2.2. Phospholipases

Phospholipases play an important role in the pathogenesis of many fungi, helping to destroy host cell membranes and penetrate tissue barriers [136,137]. In the case of *C. albicans*, high phospholipase activity correlates with strain virulence and tissue invasion ability [138]. *Rhodotorula* spp. also possesses phospholipase enzymes [28]. Furthermore, some studies suggest that *R. mucilaginosa* may produce phospholipases with even higher activity than *C. albicans*. Comparative studies have found that most *R. mucilaginosa* strains secrete phospholipases (measured on a lecithin medium)—the percentage of phospholipase-positive isolates ranged from 82 to 100%, and the enzymatic activity (hydrolysis zone diameter) was high [126,127]. For example, in one study, 82.4% of the tested *Rhodotorula* isolates showed phospholipase activity, with 69.1% of the isolated strains having strong activity (PZ coefficient < 0.7) [127]. Krzyściak (2010), on the other hand, noted that *R. mucilaginosa* at 37 °C forms larger phospholipid hydrolysis zones than the reference strains of *C. albicans* [126]. The ability to produce phospholipases may be a crucial attribute in the invasiveness of *Rhodotorula* fungi. Interestingly, *R. mucilaginosa* isolates derived from humans are more likely to exhibit phospholipase activity than those from environmental or animal sources [139]. In one study, all *Rhodotorula* spp. isolated from patients tested positive for phospholipase, while many strains isolated from, for example, bird cloacas did not produce this enzyme [139]. This suggests that phospholipase activity may be an adaptive factor associated with colonisation and infection of the human body [140]. The actual role of *Rhodotorula* spp. phospholipases in the course of infection is not yet fully understood. These enzymes may promote damage to epithelial cells, obtain nutrients from host cell membranes and facilitate the fungus in overcoming tissue barriers [141,142]. It has been demonstrated that *Rhodotorula* strains that do not produce phospholipase at 30 °C also fail to grow at 37 °C [126], suggesting a correlation between the activity of this enzyme and the ability to survive in the human body.

#### 4.2.3. Hydrolytic Enzymes

Besides phospholipases, *Rhodotorula* fungi also produces other hydrolytic enzymes that may contribute to virulence, especially proteases (proteolytic enzymes) and lipases/esterases [140,141]. Fungal proteases, especially those from the aspartyl protease (Sap) family, enable the breakdown of host proteins (e.g., collagen, elastin, immunoglobulins) and evasion of immune mechanisms [143,144,145]. *R. mucilaginosa* isolates were found to have a significant proteolytic capacity—approximately one-third of the strains tested showed protease activity on protein substrates [126]. Notably, the level of aspartyl protease activity in these protease-positive *Rhodotorula* strains was comparable to the protease activity found in the pathogenic *C. albicans* [126]. This indicates that selected *Rhodotorula* strains can achieve high proteolytic potential, which in vivo facilitates tissue destruction and avoidance of, for example, host immune proteins.

The ability to hydrolyse fats (lipolysis) is another potential virulence factor associated with carbon acquisition from complex host membrane lipids and modulation of the infection environment [146]. In the case of *Rhodotorula* spp., lipase/esterase activity appears to be less well understood – in one study, only 16.7% of *R. mucilaginosa* strains showed lipase activity at 37 °C, with a lower degree of ester hydrolysis compared to the comparative *C. albicans* strains [126]. Other *Rhodotorula* species (e.g., *R. glutinis*) may show a slightly higher frequency of esterase production—in a study by Seifi et al., 29.4% of *R. glutinis*/*R. minuta* isolates tested positive for esterases (on Tween 80 medium). In contrast, *R. mucilaginosa* isolates in this study did not produce this enzyme [127]. Overall, however, the ability of *Rhodotorula* to hydrolyse lipids appears to be limited to a minority of strains, which may distinguish them from *Candida*, where lipases are more common [127]. Nevertheless, the presence of proteolytic and lipolytic enzymes in at least part of the *Rhodotorula* population may increase their pathogenic potential by enabling them to colonise niches in host tissues and facilitating colonisation [141,146]. It is worth mentioning that biochemical tests, such as API ZYM, have shown a high similarity in the enzymatic profiles of different *Rhodotorula* strains (e.g., esterase, lipase, and leucine arylamidase activity), which, however, proved to be of little use in differentiating between strains [141,147]. From the point of view of virulence, however, it is crucial that the combined action of phospholipases, proteases, and possibly lipases can synergistically damage tissue barriers and host cells, thereby paving the way for yeasts to penetrate deeper tissues [148].

#### 4.2.4. Iron Acquisition

Obtaining iron in the host organism is fundamental to the pathogenicity of many microorganisms [149,150,151]. Yeast-like fungi often secrete haemolysins—factors capable of lysing erythrocytes, releasing haemoglobin as a source of iron [151,152]. *Rhodotorula* spp. also exhibit haemolytic activity [153]. For comparison, *Candida albicans* or *C. tropicalis* also exhibit varying degrees of haemolytic activity associated with the secretion of peptide cytolysins that bind to erythrocyte membranes [154]. The haemolytic activity of *Rhodotorula* spp. confirms that these yeasts can obtain iron from haemoglobin in a similar way to *Candida*, which probably promotes survival in the host organism, where the availability of free iron is low [153,155]. It has been shown that the haemolysin activity of *Rhodotorula* may vary between species. In a study by Seifi et al., a positive haemolysis test was obtained in 69.1% of all *Rhodotorula* isolates, with the highest percentage of haemolysin-positive isolates found in *R. glutinis* [127]. Haemolysins may facilitate fungal colonisation of blood and highly vascularised tissues by providing access to essential iron from host erythrocytes [82].

#### 4.2.5. Adhesion

Adhesion to surfaces—both inanimate (e.g., catheters, prostheses) and living (epithelial cells, endothelium)—is a precondition for colonisation and biofilm formation by yeast-like fungi [156,157]. Although *Rhodotorula* species does not produce known specific adhesins like some *Candida* species, it exhibits cell surface hydrophobicity and other characteristics that promote adhesion [22,158,159]. Studies on the closely related teleomorphic stage of *Rhodotorula* fungi (known as *Rhodosporidium toruloides*) have shown that capsular cells (having a polysaccharide capsule) were more hydrophobic and adhered more strongly to plastic surfaces than non-capsular mutants [159]. This suggests that specific components of the fugal cell wall—possibly the capsule or glucuronic components—enhance adhesion by modifying the charge and hydrophobicity of the cell [56,57]. Furthermore, it has been found that under serum growth conditions, some *R. mucilaginosa* cells produce a thin polysaccharide capsule and very rarely form short pseudohyphae, which may promote adhesion to biological surfaces [54,56]. The coat, similar to that of the related *Cryptococcus*, may perform anti-phagocytic functions and indirectly influence adhesion to host cells [57].

In vitro models have demonstrated *Rhodotorula’s* ability to adhere to synthetic materials [26,27]. Regarding adhesion to host cells, there are no detailed quantitative data; however, the presence of *Rhodotorula* spp. on the mucous membranes and skin of animals suggests that these fungi are capable of adhering to the epithelium [13,21]. Adhesion is likely mediated by non-specific hydrophobic interactions and possibly binding to extracellular matrix components (such as fibronectin or laminin), as is the case with *Candida* [27]. Phenotypic change may play a role here—it has been observed that non-adherent Rhodosporidium cells can eventually become adhesive, e.g., with the onset of budding, which suggests that the developmental phase controls adhesion [159,160]. In summary, *Rhodotorula* can colonise a variety of surfaces, especially when conditions are favourable (presence of foreign bodies, prolonged exposure). Features such as the capsule and the formation of pseudohyphae by some strains further enhance adhesion and hinder the host’s defence mechanisms from eliminating yeast [21,56,57].

#### 4.2.6. Urease Activity

*Rhodotorula* spp. can produce urease, an enzyme that breaks down urea into ammonia and carbon dioxide [53,56]. It is hypothesised that urease in *Rhodotorula* fungi also plays a role analogous to urease in *Cryptococcus* spp., facilitating nitrogen extraction from urea and alkalising the environment. However, direct functional evidence for this genus is limited [56,161] Ammonia formation raises pH, which may neutralise acidic environments and favour yeast survival in host tissues (e.g., in the acidic pH of the skin or within the phagosomes of macrophages) [161,162]. Although direct studies of *Rhodotorula* spp. mutants lacking the ability to produce urease have not been studied. By analogy, it can be cautiously assumed that this enzyme facilitates the colonisation of urea-rich niches (e.g., on the skin, where urea is present in sweat) and potentially increases invasiveness [22,162]. In *Cryptococcus neoformans*, urease is a virulence factor that determines neurotropism and damage to the vascular endothelium – deletion of the urease gene significantly reduces the virulence of this fungus and its ability to colonise the brain [161,163]. However, in *Rhodotorula* spp., the role of urease in overcoming systemic barriers has not been clearly confirmed, representing a significant research gap [22,56].

Nevertheless, all of the most studied clinical strains of *Rhodotorula* spp. demonstrated urease activity, which distinguishes them from other yeast-like fungi, including *Candid*a *albicans* and most *Candid*a spp., which do not produce urease [53,56]. This suggests that *Rhodotorula* spp. are adapted to environments with limited nitrogen sources; however, further investigation is required to determine the role of this adaptation in terms of virulence factors [22,56].

#### 4.2.7. Catalase

Another putative virulence factor is catalase (CAT), an enzyme that protects fungal cells from oxidative stress [164,165]. *Rhodotorula* spp. are aerobic organisms and exhibit high basal catalase and superoxide dismutase (SOD) activity even during growth on sugar-rich media; the levels of both enzymes depend on the culture conditions [166,167]. Under increased oxidative metabolism, such as during growth on ethanol or in response to stressors, *Rhodotorula* spp. can further enhance their antioxidant response [164,168]. In *R. glutinis*, switching from fermentation medium to ethanol has been shown to result in a further ~30–50% increase in catalase activity (and a ~10–15% increase in SOD activity) [167]. This demonstrates the ability of these fungi to adapt and neutralise excess reactive oxygen species [164,167]. Catalase breaks down hydrogen peroxide (H_2_O_2_) into water and oxygen, which is crucial in defence against host immune mechanisms based on oxygen burst [165]. Macrophages and neutrophils attack pathogens, among other ways, by producing H_2_O_2_ and other reactive oxygen species in phagolysosomes; by analogy with other yeast-like fungi, it can be assumed that the presence of catalase allows *Rhodotorula* spp. to neutralise these compounds before they can damage the fungal cell [165,169,170]. Similarly to catalase, carotenoid production increases under the influence of environmental stressors—*Rhodotorula* has been observed to increase the synthesis of these pigments under conditions of exposure to UV, high osmolarity, or the presence of peroxides [118,164]. The synergistic action of enzymatic (catalase, peroxidases, SOD) and non-enzymatic (carotenoids) antioxidant mechanisms allows *Rhodotorula* spp. to survive in a highly oxidative inflammatory environment [164,165]. In practice, this means that the phagocytosis of these fungi by leukocytes may not immediately lead to their destruction – it can be assumed that they will be able to survive inside phagolysosomes due to H_2_O_2_ neutralisation and thus avoid rapid elimination by the host’s immune system [109,117]. Nevertheless, it can be assumed that due to CAT, SOD, and carotenoids, their tolerance to ROS increases, which may delay their killing by immune cells [109].

#### 4.2.8. Carotenoids

One of the most striking features of *Rhodotorula* fungi is the production of intensely coloured pigments—carotenoids (including torulene, torularhodin and β-carotene)—which give the colonies a colour ranging from salmon to red [171,172]. Although these pigments are not typical ‘virulence factors’ in the classical sense (they do not directly damage tissues), they can affect the survival of yeast in the host organism. Carotenoids act as antioxidants – they protect yeast cells from oxidative damage caused, for example, by reactive oxygen species released by host phagocytes [119,173]. It has been shown that depriving *R. mucilaginosa* of its ability to synthesise carotenoids (e.g., through mutagenesis or the action of inhibitors) makes cells more susceptible to oxidative stress [174]. Similarly, it is believed that in the related *Cryptococcus neoformans*, the presence of melanin in the cell wall is an important virulence factor that protects against free radicals. In the case of *Rhodotorula*, the protective function may be performed by the carotenoid layer in the cell membrane/wall [174,175]. Furthermore, some observations suggest that *Rhodotorula* pigmentation may affect their sensitivity to certain drugs [32,82]. It is believed that carotenoids may interact with the cell membrane in a way that hinders the action of echinocandins, although this assumption has not been conclusively proven [82]. However, depriving *Rhodotorula* of its ability to produce pigments often results in a decrease in its overall viability and resistance to environmental factors, which indirectly indicates the protective role of the pigment [119]. In the context of interactions with the immune system, it can be assumed that *Rhodotorula* spp. carotenoids may also be less recognisable to specific host receptors or more resistant to the action of skin antimicrobial peptides [45,176]. *Rhodotorula* spp. carotenoid pigments are considered to be a factor promoting the survival of yeast in adverse conditions (oxidative stress, UV radiation, starvation), which in the host organism translates into an increased ability to survive an immune attack [22]. Although there is no clear evidence, pigmentation can be considered an element of virulence—a ‘natural antioxidant shield’ for yeast.

## 5. Drug Sensitivity and Resistance Mechanisms

Fungi of the genus *Rhodotorula* exhibit a characteristic profile of sensitivity to antifungal substances, which differs from that of commonly found yeasts of the genus *Candida* [21,35]. They are characterised by innate (natural) resistance to many commonly used antifungal agents, which has important therapeutic implications [35]. Most importantly, *Rhodotorula* spp. are naturally resistant to most azoles, especially fluconazole [32,60]. In addition, these fungi are resistant to the entire group of echinocandins (e.g., caspofungin, micafungin) [32]. On the other hand, *Rhodotorula mucilaginosa* usually remains sensitive to polyenes (amphotericin B) and flucytosine, which provides effective therapeutic options [32].

### 5.1. Resistance to Azoles

**Figure 3:**
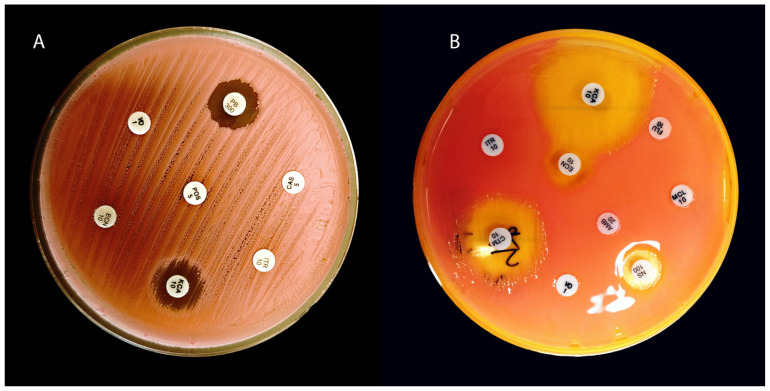
Mycograms (**A**)—*Rhodotorula mucilaginosa*; (**B**)—*Rhodotorula glutinis*. Both strains were isolated from the ear canals of dogs with OE. VO—voriconazole, PB300—polymyxin B, CAS—capsofungin, ITR—itraconazole, KCA—ketoconazole, ECN—econazole, POS—posaconazole, MCL—miconazole, CTM—clotrimazole, AMB—amphotericin B, NS—nystatin.

In in vitro studies, *R. mucilaginosa* strains were able to grow in environments with high concentrations of fluconazole (e.g., MIC50 > 128 µg/ml), confirming the lack of activity of this drug [32,177]. Clinically, this means that fluconazole is not an effective treatment option, as fungi (Figure 3) of the genus *Rhodotorula* have been reported to cause fungemia despite prophylaxis or treatment with fluconazole (so-called breakthrough infections) [23,178]. Furthermore, natural resistance to fluconazole means that long-term prophylaxis with this drug (e.g., in haematological oncology patients) may lead to the selection and colonisation of niches by *Rhodotorula* spp. after the elimination of susceptible *Candida* species from the patient’s flora (*Rhodotorula* spp. has been isolated from the blood of patients receiving continuous fluconazole treatment) [110,178].

The susceptibility of *Rhodotorula* spp. to other azoles is variable and generally limited. Itraconazole and voriconazole exhibit slightly better activity than fluconazole; however, most *Rhodotorula* strains remain classified in vitro as non-susceptible or moderately susceptible to these drugs [26,59]. In one study, as many as 95.6% of *R. mucilaginosa* isolates had an MIC ≥ 2 µg/ml for itraconazole, confirming its low efficacy against these yeasts [177]. Individual isolates (e.g., *R. glutinis*) with moderate sensitivity to ketoconazole or posaconazole have been described [35,59,179]. However, the entire azole group is generally considered clinically ineffective in treating infections. Newer triazoles (e.g., posaconazole, isavuconazole) may sometimes achieve slightly lower MIC values (greater in vitro activity than voriconazole). However, there are no clear recommendations regarding the use of azoles in treating this type of infection [35,177]. The data for isavuconazole are inconsistent (small series suggested low MICs, while more recent studies have shown high MIC50/MIC90 values for *R. mucilaginosa*) [180]. In practice, there have been cases of successful treatment of fungaemia with voriconazole, as well as numerous therapeutic failures [181]. For this reason, azoles are not recommended as first-line therapy for infections (they are only considered as last-resort drugs in cases of contraindications to amphotericin B) [181].

It is believed that fungi of the genus *Rhodotorula* may possess efflux pumps that actively expel drug molecules from the cell [182,183,184]. In pathogenic fungi, the overexpression of ABC/MFS transporters reduces intracellular azole concentrations, a key mechanism of resistance. In *Rhodotorula* spp., the efflux mechanism remains hypothetical: homologues of CDR/MDR genes and data on ABC/MFS induction by toxins have been described, but there is no direct evidence that overexpression of these pumps explains the ineffectiveness of azoles in this genus [182,184,185,186]. Additionally, it has been suggested that clinically important *Rhodotorula* species may contain less ergosterol in their cell membrane (however, this largely depends on the environmental conditions in which they currently reside) or have a slightly altered structure of the target enzyme, which reduces the effectiveness of azoles [187,188].

### 5.2. Resistance to Echinocandins

*Rhodotorula* spp. exhibits natural resistance to the entire group of echinocandins (e.g., caspofungin, micafungin) [26,35]. In vitro, very high MIC values are observed—e.g., MIC50 for caspofungin >8 µg/ml, indicating that these substances do not effectively inhibit the growth of these fungi [32]. The mechanism of this primary resistance is most likely due to the structure of the cell wall, which, like that of related fungi of the genus *Cryptococcus*, contains low levels of 1,3-β-D-glucan [82,189]. Structural differences in the glucan synthase complex are also possible, which further reduces sensitivity to drugs of this class [190]. As a result, echinocandins are ineffective, which in practice means they should not be used in cases of suspected infection caused by *Rhodotorula* spp. [30,35]. Furthermore, some data suggest that carotenoid pigments may interfere with echinocandins (e.g., through photoprotective or antioxidant effects), although there is no direct evidence to support this [82,191].

### 5.3. Sensitivity to Polyenes and Flucytosine

Species belonging to the genus *Rhodotorula* usually remain sensitive to amphotericin B—almost all strains have low MIC values for this drug [32,34]. Studies have reported that the MIC50 for amphotericin B is approximately 0.5–1 µg/ml, and even the least sensitive isolates rarely exceed 2 µg/ml [32,34]. Importantly, no resistance to amphotericin B has been observed in vivo [30,35]. The mechanism of action of amphotericin B (damage to the cell membrane through ergosterol binding) effectively destroys fungal cells, making this drug fungicidal and the treatment of choice for serious infections [35,192].

These fungi are also sensitive to flucytosine—most strains of *R. mucilaginosa* are susceptible to it, although resistant strains have also been observed after prior exposure. [32,34,193]. For example, in a 2025 study, as many as 90.5% of *R. mucilaginosa* isolates were sensitive to 5-FC, and approximately 9.5% were resistant [13]. For this reason, 5-FC is recommended for use in combination with amphotericin B, especially in severe infections, similar to the treatment of cryptococcosis [35,194]. Monotherapy with flucytosine is not recommended—there is a high risk of selecting resistant mutants during treatment [194].

## 6. Veterinary Cases

Infections caused by fungi of the genus *Rhodotorula* in animals are rare; however, isolated clinical cases in various species have been reported in the literature [21,97,195]. These infections are usually opportunistic in nature, occurring in weakened animals (with immunosuppression, following prolonged antibiotic therapy) or in the presence of concomitant bacterial or parasitic infections [97,195]. Selected veterinary cases are presented in Table 3.

These fungi can cause a variety of infections in animals, ranging from superficial skin infections to deep organ infections [195,198,199,200]. Most contemporary descriptions pertain to dogs; clinical cases in cats are sporadic [21,97,196,203]. Pressler noted that in dogs, *Rhodotorula* is sometimes isolated as an opportunistic infectious agent (so-called rhodotorulosis) in situations of immunosuppression or chronic disease, although these infections are rare overall [204]. Biegańska et al. described the first case of respiratory tract infection in a dog involving *R. mucilaginosa*, which co-occurred with *Trichosporon jirovecii* and bacteria (*Pseudomonas aeruginosa*, *Escherichia coli*) in a dog suffering from bronchotracheitis [97]. This animal had numerous predisposing factors (hypothyroidism, long-term steroid treatment), and after combination therapy (antibiotics + antifungal drugs), clinical improvement and negative control cultures were achieved [97]. Another description concerns a dog with an epididymal granuloma. From tissue of the affected epididymis, collected after castration, *Rhodotorula glutinis* was cultured—this was the first report of such a location of infection from *Rhodotorula* spp. in a dog [196].

**Figure 4:**
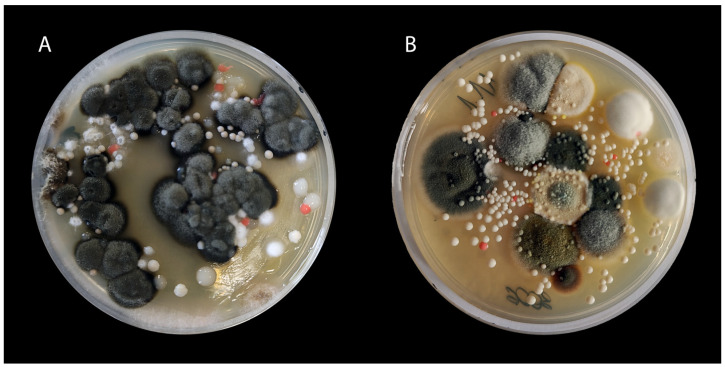
(**A**,**B**)—Sample mycological cultures from the ears of dogs with OE. Both cases show heavy mould contamination and growth of yeast-like fungi, including *Rhodotorula* species.

In cats, fungi of the genus *Rhodotorula* have been sporadically reported as the cause of dermatomycosis [21,197]. The isolation of *R. mucilaginosa* from chronic inflammatory lesions of the external auditory canal in domestic cats has also been reported. However, it was usually considered an environmental contaminant or a component of mixed flora [203]. In a study by Aboul-Ella et al., *R. mucilaginosa* was isolated from 4.7% (21/450) of all clinical samples; isolates were obtained from, among others, the ear canal and nasal cavities of dogs and cats with otitis externa or rhinitis [13]. This suggests that these fungi may contribute to the development of ear inflammation in small animals, particularly in the context of mixed infections (Figure 4) with bacteria [13,203].

Fungi from genus *Rhodotorula*, in farm animals, are also described sporadically [201,205]. Dworecka-Kaszak et al. studied yeasts isolated from the milk of cows suffering from mastitis; fungi of the genus *Candida* predominated among the isolates. However, *Rhodotorula* spp. were also detected in single samples [206]. Wawron et al. described cases of yeast mastitis in dairy cows—alongside *Candid*a spp., fungi of the genus *Rhodotorula* were also found as one of the components of mixed infections [207]. Furthermore, the literature mentions cases of pneumonia in sheep caused by *R. mucilaginosa* [200]. *Rhodotorula* spp. was also isolated from the ear canals of cattle during an invasion of *Raillietia* mites; these fungi colonised the inflamed ear, subsiding after the parasitosis was cured [195]. In poultry, two cases of skin lesions have been described: one in chickens, where *Rhodotorula mucilaginosa* was identified as the causative agent, and the other in broiler dermatitis caused by *Rhodotorula glutinis*. This suggests that these fungi may act as cutaneous pathogens in poultry [208,209]. Alvarez-Pérez et al. reported an unusual case of skin lesions in a Patagonian fur seal in the Madrid Zoo, caused by *R. mucilaginosa* [198].

## 7. Conclusions and Future Directions

*Rhodotorula* spp. are often under-recognised, yet they are clinically significant opportunistic pathogens that can affect both animals and humans. In laboratory and clinical practice, these fungi should not be dismissed as mere “contaminants,” especially in cases that are associated with disease, such as inflammatory lesions, positive cytology, abundant or repetitive growth, or isolation in monoculture. In such contexts, *Rhodotorula* species may play a role as an etiological factor. From a veterinary perspective, it is essential to maintain a close microbiological-clinical correlation to differentiate between colonisation and infection. This is critical to avoid both overdiagnosis and underestimation of the pathogen. Improving our understanding of *Rhodotorula* spp. is crucial to developing more effective prevention and treatment strategies for the rare but serious infections it can cause. Consequently, a better grasp of *Rhodotorula* species as potential pathogens will help in interpreting laboratory results and lead to more effective, evidence-based therapeutic management, particularly in the presence of coexisting risk factors.

## Figures and Tables

**Table 1:** Selected biochemical features helpful in identification and differentiation.

Feature/Test	*Rhodotorula* *mucilaginosa*	*Rhodotorula* *glutinis*	*Cryptococcus* *neoformans*	*Candida* *albicans*
Nitrate (NO_3_^−^) assimilation	No—does not assimilate	Yes—present (requires specific culture conditions)	No—does not assimilate	No—does not assimilate
Growth at 37 °C	Yes—grows (some strains up to 40 °C)	Weak or absent—variable (usually limited)	Yes—grows at 37 °C	Yes—grows (many strains up to 42 °C)
Tolerance to 0.1% cycloheximide	No growth—susceptible	Variable—some strains grow weakly	No growth—susceptible	No growth—susceptible (most strains)
Maltose assimilation	Variable—strain-dependent	Positive—assimilates	Positive—assimilates	Positive—assimilates
Raffinose assimilation	Positive—assimilates raffinose	Variable—strain-dependent	Variable—most strains positive, some weakly	Negative—does not assimilate
Melezitose assimilation	Weak or strain-dependent	Positive—clearly assimilates	Positive—assimilates melezitose (typical trait)	Variable—strain-dependent (partial)
D-glucosamine assimilation	Variable/weak—partial	Negative—does not assimilate this sugar	Negative—does not assimilate this sugar	Positive
Erythritol assimilation	Variable—strain-dependent	Negative—does not assimilate	Negative—does not assimilate	Negative—does not assimilate
Urease (urea hydrolysis)	Positive—urease-producing	Positive—urease-producing	Positive—strong urease activity (test positive)	Negative—no urease
Catalase	Positive—present	Positive—present	Positive—present	Positive—present
Hemolysis (blood agar)	Variable—many strains show β-hemolysis (enhanced at 37 °C)	Variable—β-hemolysis reported in some strains (weaker than *R. mucilaginosa*)	Negative—no hemolysis (no hemolysins)	Positive—hemolysis present
Myo-inositol assimilation	Negative—does not assimilate	Negative—does not assimilate	Positive—assimilates inositol	Negative—does not assimilate
Colony pigmentation	Pink to coral, smooth, often mucoid	Salmon-pink to orange; smooth or wrinkled (glossy to matte)	Cream to white, mucoid; dark brown on Staib medium (melanin)	White to cream, smooth, shiny

**Table 2:** Comparison of diagnostic methods — advantages, limitations, time to results, and typical applications.

	Advantages	Limitations	Turnaround Time	Typical Applications	Citations
Culture (SDA/YGC)	recovery of live pure isolate; low cost and wide availability; starting point for MALDI/PCR/AFST; pigmented colonies support genus-level suspicion	colony appearance ≠ species ID; potential confusion with other “red yeasts”; full pigmentation delayed	colonies: 48–72 h; full pigmentation: 72–120 h	routine plating; obtaining pure culture for downstream testing	[30,67,100,101]
Chromogenic media	rapid detection of mixed cultures; visual segregation of yeasts by color	platforms designed for *Candida* spp.; for *Rhodotorula* spp. colors are non-specific; growth inhibited on some media	typically 48 h readout	screening for yeasts -like fungi	[102,103]
Direct microscopy	immediate result; assessment of clinical relevance (yeast cells + inflammatory context); very low cost	no species ID; lower sensitivity in cell-poor specimens; expertise required	minutes	triage of ear/skin specimens; rapid yeast confirmation	[31,84]
Biochemical tests (API, Vitek)	commercial panels; genus/species ID for common yeasts; no specialized instrumentation	lower accuracy for rare yeasts; “no-ID”/mis-ID possible; time-consuming	+18–72 h post-isolate (API ~48–72 h; Vitek ~18–24 h)	settings without MALDI/PCR; genus-level verification	[104,105,106]
MALDI-TOF MS	rapid species-level ID after colony; low per-test cost; high throughput; libraries continuously updated	requires pure culture; library gaps for very rare species; indeterminate results need confirmation	hours (same day as colony)	routine and urgent ID; surveillance/epidemiology	[104,107]
PCR + sequencing (ITS)	highest specificity; resolves rare/spurious cases; PCR directly from specimen possible	cost/know-how; sequence analysis required; not always within 1 day	24–72 h (≈24 h expedited)	species confirmation; taxonomy/phylogeny; when culture is negative	[30,51,108]
Histopathology (H-E, PAS, GMS)	evidence of tissue invasion; distinguishes colonization vs. infection; assesses complications (e.g., endocardial involvement)	species ID usually not possible; tissue required; correlation with culture/MALDI/PCR needed	frozen: hours; routine: 24–48 h	suspected IFD; endocarditis/tissue lesions	[30,31]
AFST (MIC; EUCAST BMD)	quantitative in vitro susceptibility profile; aids severe/failing cases; surveillance data	no EUCAST clinical breakpoints for *Rhodotorula* spp.; adds 1–2 days to TAT; cost/labor of reference methods	+24–48 h post-ID	therapy selection in severe infections; research/monitoring	[82,109]

ITS: Internal Transcribed Spacer; H-E: Hematoxylin–Eosin; PAS: Periodic Acid–Schiff; GMS: Grocott’s Methenamine Silver; AFST: Antifungal Susceptibility Testing; MIC: Minimum Inhibitory Concentration; EUCAST: European Committee on Antimicrobial Susceptibility Testing; BMD: Broth Microdilution; ID: Identification (genus/species); TAT: Turnaround Time; IFD: Invasive Fungal Disease; ≠—not equivalent; ≈—approximately; post-isolate/post-ID—additional time counted from obtaining a pure isolate or from completing identification.

**Table 3:** Selected veterinary cases.

Case (Animal Species)	*Rhodotorula* Species	Therapy and Treatment Outcome	Citation
Dog, 6 years (male, mixed breed)—chronic tracheobronchitis	*R. mucilaginosa*	Marbofloxacin (antibiotic) + azole antifungal (Canizol^®^); clinical improvement; follow-up cultures negative (complete cure).	[97]
Dog, 4 years (male, mixed breed)—scrotal lesions; epididymitis	*R. glutinis*	Surgical removal of the affected epididymides; no detailed data on antifungal therapy—case cured surgically.	[196]
Cat, 3 years (female)—chronic skin lesions	*R. mucilaginosa*	No complete treatment data; skin lesions resolved after topical azole (ketoconazole), according to the case authors.	[197]
South American fur seal (female, in a zoo)—skin lesions on the trunk	*R. mucilaginosa*	Topical azole therapy (sertaconazole) for several weeks; marked improvement and healing of lesions (confirmed by negative follow-up testing).	[198]
Cow—otitis externa with mite infestation	*R. mucilaginosa*	No specific treatment targeting *Rhodotorula* spp. (therapy was directed against parasites and bacteria); yeasts eliminated after addressing the primary cause.	[195]
Chickens (Uganda)—necrotic skin lesions	*R. mucilaginosa*	No treatment data (descriptive outbreak report).	[199]
Sheep—mycotic pneumonia	*R. mucilaginosa*	No treatment data (case report—infection diagnosed at necropsy after death).	[200]
Cow—mycotic mastitis	*R. mucilaginosa*	No data on specific antifungal therapy (cases noted in a study of dairy herds; mastitis treatment not described in the context of *Rhodotorulav* spp.).	[201]
Laboratory rats (experimental model)—disseminated fungal infection (generalized rhodotorulosis)	*R. mucilaginosa*	No treatment (experimental model under immunosuppression; severe lesions observed in internal organs—lungs, liver and spleen—caused by the infection).	[202]

## Data Availability

Not applicable.
